# Effects of silymarin on p65 NF-κB, p38 MAPK and CYP450 in LPS-induced hoof dermal inflammatory cells of dairy cows

**DOI:** 10.1186/s12917-019-1868-y

**Published:** 2019-04-30

**Authors:** Meng-Yue Tian, Jing-Hui Fan, Zhi-Wei Zhuang, Fei Dai, Cheng-Yu Wang, Hai-Ting Hou, Yu-Zhong Ma

**Affiliations:** 1 College of Veterinary Medicine, Hebei Agricultural University, 2596 Lekai South Street, Baoding, 071001 Hebei China; 2 Shandong New Hope Liuhe Co. Ltd, Qingdao, 266000 Shandong China

**Keywords:** Silymarin, Dairy cow, Hoof dermal cell

## Abstract

**Background:**

Laminitis is considered as one of the most important causes of hoof lameness in dairy cows, which can lead to enormous economic losses. However, the etiology and pathogenesis of laminitis have not been clarified yet. Besides, it is of great significant to find alternative herbs for the prevention and treatment of dairy hooves to avoid the antibiotic abuse. In this study, the primary hoof dermal cells of dairy cows were isolated, the inflammatory model was induced by LPS, and treated with silymarin to find whether silymarin has protective effect on the inflammatory dermal cells. The viability of dermal cells, the levels of IL-1β and TNF-α, the degree of p65 NF-κB and p38 MAPK phosphorylation, the expressions of CYP3A4 and CYP1A1 were measured.

**Results:**

Hoof dermal cells of dairy cows were successfully isolated and cultured by tissue adherent culture method. Certain concentrations of LPS can increase the levels of IL-1β and TNF-α, promote the phosphorylation of p65 NF-κB and p38 MAPK, and inhibit the mRNA expressions of CYP3A4 and CYP1A1. The optimal concentration for LPS to establish a hoof dermal cells inflammatory model was 10 μg/mL. Certain concentrations of silymarin can markedly decrease the secretions of IL-1β and TNF-α, inhibit the phosphorylation of p65 NF-κB and p38 MAPK, and promote the mRNA expressions of CYP3A4 and CYP1A1 in LPS-induced dermal inflammatory model.

**Conclusions:**

LPS can be used for inducing the hoof dermal cells inflammatory model of dairy cows. Silymarin has protective effects on the LPS-induced inflammatory model.

## Background

Hoof disease is one of the major diseases of dairy cows, among which laminitis is the most common one [1]. Laminitis is defined as a diffuse aseptic inflammation of corium in hoofed animals [2], which can lead to many kinds of claw horn lesions, including sole ulcers, white line disease and sole hemorrhage of dairy cows [3]. Economic losses caused by laminitis were enormous, which can severely restrict the development of dairy farming. In recent years, many scholars have focused on the pathophysiology [2], histology [4], metabolomics [5] and proteomics [6] of dairy cow laminitis, but the etiology and the pathogenesis have not been clarified yet. In previous studies, the acute laminitis model in vivo had been established successfully [7], whereas the interference caused by various physiological factors in vivo cannot be ignored, so there is still a high demand to establish an in vitro model for further exploration of laminitis.

Endotoxins, also known as lipopolysaccharides (LPS), is a major component of the outer membrane of Gram-negative bacteria. When the bacterial structure is destroyed, LPS will release into the blood and trigger a series of inflammatory reactions [8]. It showed that inflammatory reactions were the main pathological changes in early stages of laminitis [9]. Endotoxin is one of the key factors causing laminitis. For example, LPS concentrations increased in the serum of horses during oligofructose-induced laminitis model [10]. Intradermal injection of endotoxins into cattle induced a series of symptoms of laminitis [11]. Forelimbs exposed to LPS showed significant morphological changes in lamellar tissues and led to metabolic changes [12]. Therefore, stimulating the hoof dermal cells with LPS to establish an inflammatory model may be a novel way to explore the pathogenesis of laminitis.

Silymarin, a standardized extract of milk thistle, is a flavonoid, which is proved to have functions such as protecting liver [13], anti-oxidation [14], anti-inflammatory [15] and anti-cancer [16]. Clinical acute toxicity tests had demonstrated that silymarin generally had extremely low toxicity and no side effects on animal and human applications in certain dosage range [17]. In addition, previous studies have shown that silymarin could reduce endotoxin activities and improve the integrity of equine lamellar explants [18]. However, there is no scientific literature available to elucidate whether silymarin exhibits positive effects on bovine laminitis.

In this study, hoof dermal cells of dairy cows were isolated and cultured, the inflammatory model was established by LPS, and the inflammatory cells were treated with silymarin. The levels of IL-1β and TNF-α, the phosphorylation of p65 NF-κB and p38 MAPK, and the expressions of CYP3A4 and CYP1A1 were determined to study whether silymarin had protective effects on the inflammatory dermal cells, and provide a theoretical basis for herbal medicine protection on laminitis of dairy cows.

## Methods

### Materials

LPS was purchased from Sigma (St. Louis, USA). Silymarin was purchased from Yihe Co. Ltd. (Xian, China). ELISA kits of bovine IL-1β and TNF-α were purchased from DG Biotech Co. Ltd. (Beijing, China). Antibodies against phosphor-p65 NF-κB, p65 NF-κB, phosphor-p38 MAPK, p38 MAPK and GADPH were purchased from Anyan trade Co. Ltd. (Shanghai, China). Total RNA extraction kit and reverse transcription kit were purchased from CW Biotech Co. Ltd. (Beijing, China). Fluorescence quantitative real time PCR kit was purchased from TransGen Biotech Co. Ltd. (Beijing, China). RT-PCR primers were synthetized by Sangon Biotech Co. Ltd. (Shanghai, China). RIPA cell lysis buffer, HE staining kit and BCA protein concentration determination kit were purchased from Solarbio (Beijing, China).

### Isolation and culture of hoof dermal cells

Hoof tissues were obtained from adult healthy dairy cows at a commercial slaughter house. After shaved, cleansed and washed with 75% ethanol, the collected tissues were put into sterile saline solution with penicillin-streptomycin and transported on ice to the laboratory.

After rinsing with PBS and removing the subcutaneous connective tissues, the tissue blocks were trimmed into small pieces and incubated with 0.25% trypsin solution overnight at 4 °C. Then, epidermis was separated and removed, and the dermis pieces were incubated in 6-well plates coated with rat tail collagen. DMEM medium supplemented with 15% fetal bovine serum (FBS), 0.025 M HEPES, 1 × Insulin-Transferrin-Selenium (ITS) and Gentamicin was used as cultivation medium. 500 μL medium was pipetted carefully onto the tissue pieces to avoid flooding. 24 h later, when tissue pieces were attached to the plates, further 2 mL medium was added. Medium was changed every 3 days. Tissue pieces were removed 10 days later. Upon 80–90% confluency, the cells were detached with 0.25% trypsin-EDTA solution and seeded into 25 cm^2^ flasks.

For morphological staining, the cells were seeded on cover glass and stained by the HE staining kit, according to the manufacturer’s instructions.

### Cell viability

Hoof dermal cells were seeded into 96-well plates with a density of 1 × 10^5^ cells per well. After incubation with gradient concentrations of LPS or silymarin for 48 h, 20 μL 5 mg/mL MTT was added per well. 4 h later, 100 μL DMSO was added. After vibrated for 5 min, the absorbance value was measured by microplate reader.

### Treatment of the LPS-induced inflammatory cells with Silymarin

The silymarin-DMSO solution was diluted with DMEM medium and filtered with 0.22 μm microfilter. The LPS-induced inflammatory hoof dermal cells were treated with gradient concentrations of silymarin for 24 h and 48 h, respectively.

### ELISA measurement of IL-1β and TNF-α

The contents of IL-1β and TNF-α in cell supernatants of the control group, the LPS treated groups, and the silymarin treated groups were detected by ELISA kit, respectively, according to manufacturer’s instructions.

### Western blot analysis of p65 NF-κB and p38 MAPK phosphorylation

The total proteins were obtained by RIPA cell lysis buffer, quantified by BCA protein concentration determination kit, and then denatured, run in 12% SDS-polyacrylamide gel and electrotransferred to nitrocellulose membranes. The membranes were blocked with 5% non-fat milk for 1 h at room temperature, incubated with antibodies against p65 NF-κB, phosphor-p65 NF-κB, p38 MAPK, phosphor-p38 MAPK or GADPH at 4 °C overnight. After three times washing with TBST, the membranes were incubated with horse-radish peroxidase (HRP)-conjugated anti-rabbit IgG (H + L) secondary antibody (1:2000 in TBST) for 1 h at room temperature. After a final series of washing, the membranes were placed into NBT/BCIP chromogen kit for development. Relative intensity of each protein band/sample was quantified using Image J software.

### CYP3A4 and CYP1A1 mRNA expressions by RT-PCR analysis

Total cellular RNA was obtained using RNA extraction kit. After evaluated by NanoDrop 2000 spectrophotometer, the total RNA was purified and reversed into cDNA using reverse transcription kit, according to the manufacturer’s instructions. The resulting cDNA was used to detect the relative expression levels of CYP3A4 and CYP1A1 by fluorescence quantitative real time PCR. Primers used for amplification were: CYP3A4, 5′- GGAAACCTGGGTTCTCCTGGCT- 3′ (forward), and 5′-CCGATGGACCAAAAACCCTCCG- 3′ (reverse); CYP1A1, 5′-GACCTGAATCAGAGGTTCTACGTCT- 3′ (forward), and 5′-CCGGATGTGACCCTTCTCAA- 3′ (reverse); GADPH, 5′-ATGGAGAAGGCTGGGGCTCACT-3′ (forward), and 5′-AGTCCCTCCACGATGCCAAAGT-3′ (reverse). The reaction volumes were 20 μL (2× TransStart® Top Green qPCR SuperMix 10 μL, 10 μM forward primer 0.4 μL, 10 μM reverse primer 0.4 μL, template 1 μL, water 8.2 μL). PCR was performed as follows: template denaturation at 95 °C for 5 min, and 30 cycles of denaturation at 95 °C for 30 s, primer annealing at Tm-3 °C for 30 s, and primer extension at 72 °C for 30 s, followed by a final extension at 72 °C for 5 min. The relative expression level for each gene was calculated using the 2^-ΔΔCt^ method and GADPH was used as an reference gene.

### Statistical analysis

Data were presented as mean ± SD of at least three separate experiments. Significant differences among groups were determined by one-way analysis of variance (ANOVA) with Duncan’s post hoc test using SPSS 19.0 software (SPSS, Inc., Chicago, IL). A two-tailed *P*-value below 0.05 was considered as statistically significant.

## Results

### Configuration of bovine hoof dermal cells

**Fig. 1:**
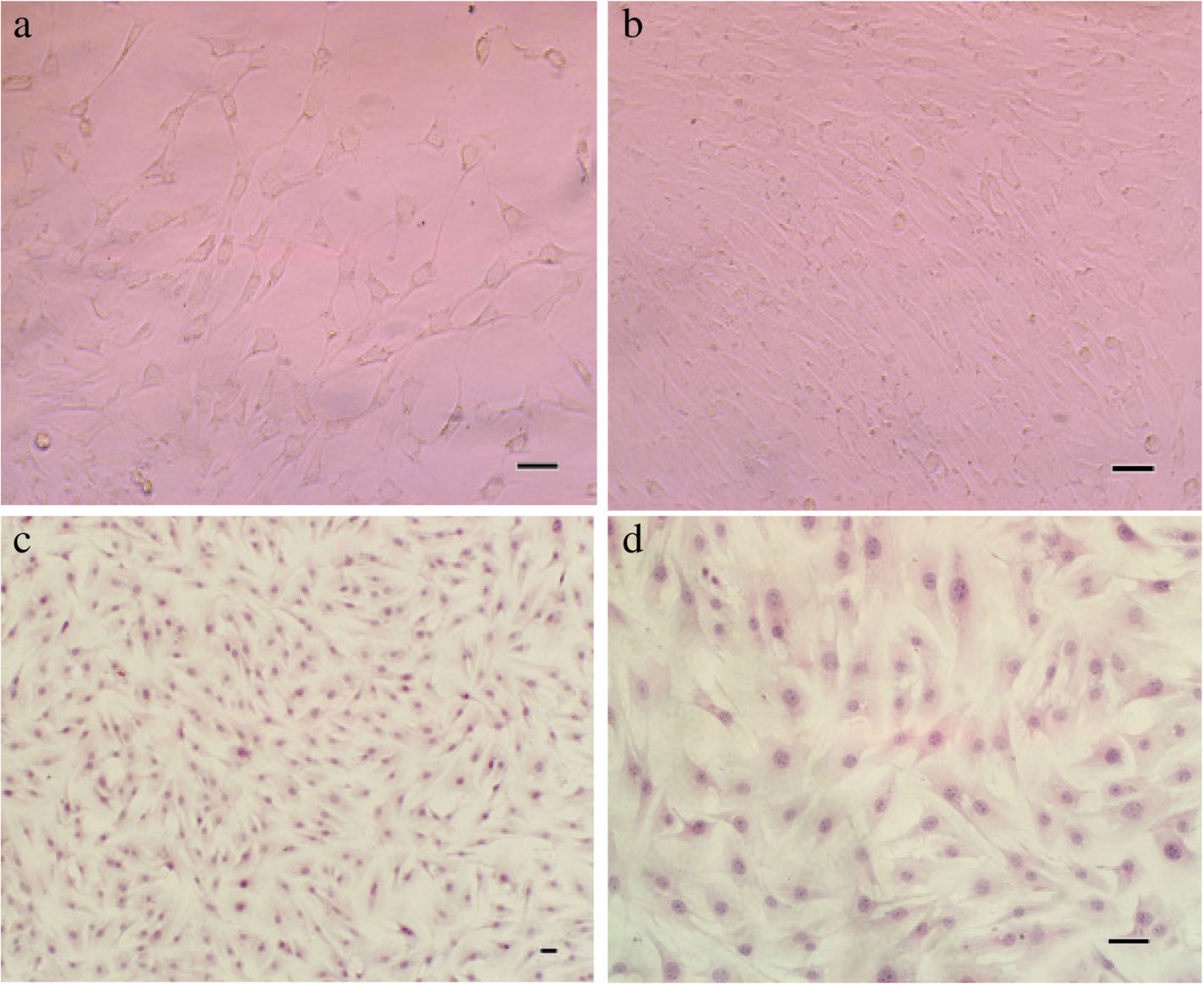
Representative photographs of bovine hoof dermal cells morphological analysis. **a**. After seeding of the tissue blocks, cells started to proliferate, showing an irregular triangle or fusiform shape; **b**. After inoculation for 15 days, dermal cells were mostly fusiform, with a certain flow direction; **c**, **d**. The sub-cultured dermal cells after HE staining were irregular in shape, polygonal or long fusiform. (scale = 100 μm)

The primary bovine hoof dermal cells isolated from tissue blocks cultured slowly. Observed under inverted microscope, dermal cells began to climb out from the edge of tissue blocks after 5–7 days of inoculation (Fig. 1a). The adherent cells approached confluence and could be sub-cultivated after incubation for 15–20 days (Fig. 1b). For HE staining, the sub-cultured cells grew well and could be used for the following experiment (Fig. 1c, d).

### Selection of the optimal concentration of LPS for the inflammatory model

**Fig. 2:**
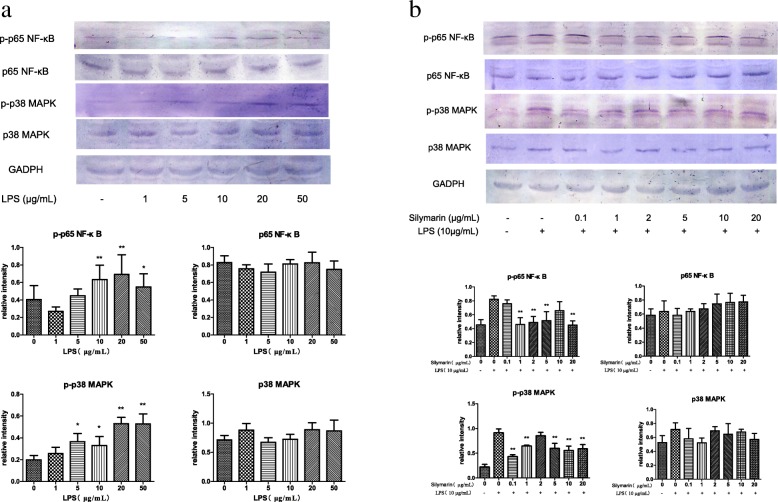
The phosphorylation of p65 NF-κB and p38 MAPK by Western blot analysis. **a**. With LPS treatment for 48 h in hoof dermal cells, the expressions of p-p65 NF-κB, p65 NF-κB, p-p38 MAPK and p38 MAPK were detected (*n* = 8). (^**^P-value< 0.01; ^*^P-value< 0.05, compared with the control group). **b**. After treated with silymarin for 48 h in LPS-induced inflammatory cells, the expressions of p-p65 NF-κB, p65 NF-κB, p-p38 MAPK and p38 MAPK were detected (*n* = 8). (^**^P-value< 0.01; ^*^P-value< 0.05, compared with the LPS model group)

**Fig. 3:**
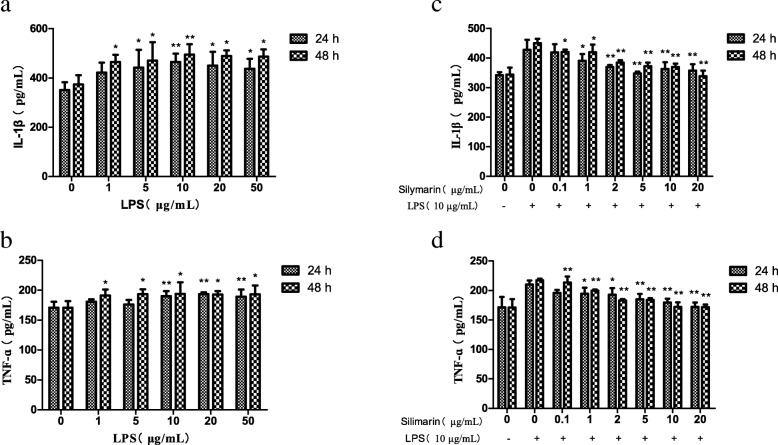
The levels of IL-1β and TNF-ɑ measured by ELISA. After treated with gradient concentrations of LPS in hoof dermal cells for 24 h and 48 h, levels of IL-1β (**a**) and TNF-ɑ (**b**) were detected (n = 8). (^**^P-value< 0.01; ^*^P-value< 0.05, compared with the control group). After treated with gradient concentrations of silymarin in LPS-induced hoof dermal inflammatory cells for 24 h and 48 h, levels of IL-1β (**c**) and TNF-ɑ (**d**) were detected (n = 8). (^**^P-value< 0.01; ^*^P-value< 0.05, compared with the LPS model group)

**Fig. 4:**
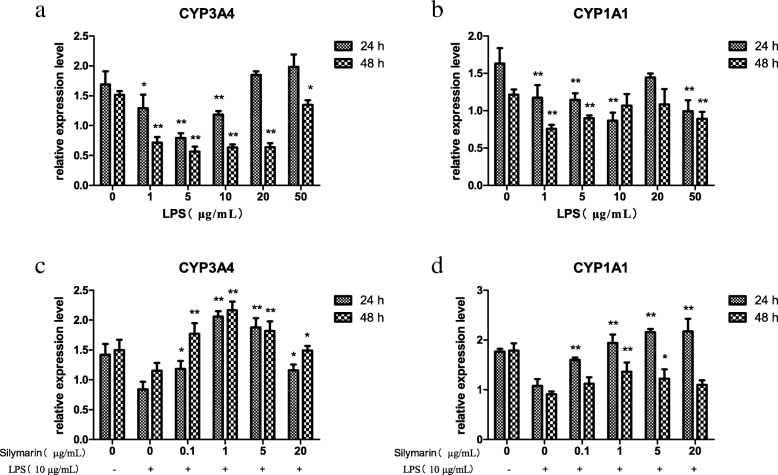
CYP3A4 and CYP1A1 expressions measured with qRT-PCR. With LPS treatment for 24 h and 48 h in hoof dermal cells, the expressions of CYP3A4 (**a**) and CYP1A1 (**b**) were detected (*n* = 8). (^**^P-value< 0.01; ^*^P-value< 0.05, compared with the control group). After treated with silymarin for 24 h and 48 h in LPS-induced inflammatory cells, the expressions of CYP3A4 (**c**) and CYP1A1 (**d**) were detected (n = 8). (^**^P-value< 0.01; ^*^P-value< 0.05, compared with the LPS model group)

The inhibition ratio (IR) increased with the gradient concentrations of LPS treatment, 10 μg/mL group was the nearest to 10% (Table 1). The phosphorylation of p65 NF-κB and p38 MAPK increased with the treatment of LPS for 48 h. For the expressions of p-p65 NF-κB, 10, 20, 50 μg/mL LPS groups were significantly different with that in the control group (*P* < 0.05), and the expressions of p-p38 MAPK increased significantly with the treatment of the 5, 10, 20, 50 μg/mL LPS (*P* < 0.05) (Fig. 2a). The IL-1β secretions increased first and then decreased with the gradient concentrations of LPS treatment, reaching a peak at 10 μg/mL, which was extremely significantly different with that in the control group (*P* < 0.01) (Fig. 3a). After treatment with 10 μg/mL LPS for 24 h and 48 h, the secretion of TNF-α was significantly different with that in the control group (*P* < 0.05) (Fig. 3b). The expressions of CYP3A4 significantly decreased with the treatment of 1, 5, 10 μg/mL LPS for 24 h, and 1, 5, 10, 20, 50 μg/mL LPS for 48 h (*P* < 0.05) (Fig. 4a). The expressions of CYP3A4 extremely significantly decreased with the treatment of 1, 5, 10, 50 μg/mL LPS for 24 h, and 1, 5, 50 μg/mL LPS for 48 h (*P* < 0.01) (Fig. 4b). From the above, 10 μg/mL LPS was the optimal concentration for inducing the inflammatory model of hoof dermal cells.

**Table 1:** The inhibitory effect of LPS on dermal cells(*n* = 8)

LPS (μg/mL)	Absorbance value	IR(%)
0	0.865 ± 0.074	0
1	0.842 ± 0.050	2.61
5	0.803 ± 0.052	7.10
10	0.772 ± 0.050^*^	10.73
20	0.702 ± 0.025^**^	18.80
50	0.622 ± 0.036^**^	28.08

Note: Cells were treated with gradient concentrations of LPS for 48 h, and the viability was monitored by MTT assay. Value indicated are the mean ± SD (^**^P-value< 0.01; ^*^P-value< 0.05). The inhibition ratio (IR) = 1- (the test group OD value/ the control group OD value)

### Expressions of p65 NF-κB, p38 MAPK and CYP450 with gradient silymarin treatment

Independent of the concentration, silymarin had no influence on the viability of hoof dermal cells (Table 2). Compared with the LPS model group, the phosphorylation of p65 NF-κB and p38 MAPK decreased with the treatment of silymarin for 48 h. The inhibition effects of p65 NF-κB phosphorylation were extremely significant in the 1, 2, 5 and 20 μg/mL groups (*P* < 0.01) (Fig. 2b). The phosphorylation of p38 MAPK decreased extremely significant with the 0.1, 1, 5, 10, 20 μg/mL silymarin treatment (*P* < 0.01) (Fig. 2b). With the gradient concentrations of silymarin treatment, the secretions of IL-1β and TNF-α decreased compared with the LPS model group. There were significant differences between silymarin groups and the model group (*P* < 0.05) except the 0.1 μg/mL treatment for 24 h (*P* > 0.05) (Fig. 3c, d). Regardless of concentrations, the expressions of CYP3A4 increased significantly with the silymarin treatment for 24 h and 48 h, compared with the LPS model group. (*P* < 0.05) (Fig. 4c). The expressions of CYP1A1 significantly increased with the treatment of 0.1, 1, 5, 20 μg/mL silymarin for 24 h, and 1, 5 μg/mL silymarin for 48 h (*P* < 0.05) (Fig. 4d). Above all, silymarin had protective effects on the LPS-induced hoof dermal cells inflammatory model, and 1 μg/mL silymarin was the optimal concentration.

**Table 2:** The toxic effects of Silymarin on dermal cells(*n* = 8)

Silymarin (μg/mL)	Absorbance value
0	0.841 ± 0.033
1	0.842 ± 0.040
5	0.841 ± 0.036
10	0.839 ± 0.037
20	0.843 ± 0.045

Note: Cells were treated with gradient concentrations of silymarin for 48 h, and the viability was monitored by MTT assay. Absorbance value was expressed as mean ± SD

## Discussion

Laminitis is the major cause of lameness in dairy cows, leading to serious economic loss. However, the etiology and pathogenesis of laminitis have not been clarified yet. Establishment of an in vitro model may be a novel way to solve the problem.

The common way for dermal cells isolation and culture is enzymatic digestion, which is frequently used in young animals such as embryonic rats [19] and fetal calves [20]. However, after digested with 1 g/L and 2 g/L type I collagenase in 37 °C for 2–4 h, no viable dermal cells were obtained. Various factors might be responsible for the failure, such as the type of enzyme, concentration of enzyme and digestion time. Therefore, further researches are needed to figure out whether hoof dermal cells of dairy cows could be isolated by enzymatic digestion. Tissue blocks for dermal cells culture are usually taken from the skins behind ears [21] and shoulders [22]. Hendry established a short-term culture method for bovine hoof explants, but did not obtain the dermal cells [23]. Based on the previous studies, the tissue blocks were firstly incubated in 0.25% trypsin solution, separated from the epidermis, and then cultured in plates in this experiment. Although dermal cells grew slowly by this method, the operation was simple and convenient, and the damage of the enzyme was alleviated, the interaction between cells could be preserved well.

Once entering the body, LPS will firstly bind with LBP and CD14 to form a ternary complex, which can be recognized by TLR4. Then the complex will activate MyD88-dependent pathway and MyD88-independent pathway, initiate intracellular signaling, activate NF-κB and MAPK signaling pathways, and finally trigger a series of pathophysiological reactions [24]. Nuclear factor-κB (NF-κB), also known as nuclear transcription factor, is the junction of multiple signal transduction pathways, heterodimer p50-p65 is its common form [25]. When activated by external stimulations like LPS, NF-κB will be released from the NF-κB-IκBα complex, transferred into nucleus, to initiate transcription of downstream genes [26]. Besides, mitogen-activated protein kinase (MAPK) is also an important signaling pathway of cells. Its subfamily p38 MAPK has the closest relationship with the inflammatory response induced by LPS [27]. NF-κB and MAPK signaling pathways play a key role in the immune response, inflammatory response, stress response, cell proliferation and apoptosis, etc. [28, 29]. In some studies it showed that there was a time-dependent increase in platelet p38 MAPK phosphorylation in the horse laminitis model induced by oligofructose, demonstrating that LPS activates platelets, leading to activation of leukocytes, and initiates the early inflammatory changes of acute laminitis [11]. In this experiment, the phosphorylation of p65 NF-κB and p38 MAPK increased after LPS treatment, which was consistent with previous studies. The secretions of IL-1β and TNF-α increased with the gradient concentrations of LPS treatment, reaching a peak at 10 μg/mL, which was significantly different from the control group. It have shown that in equine laminitis models induced by carbohydrate [30] and black walnut overload [31], the levels of inflammatory factors such as TNF-α, IL-1β and IL-6 increased, indicating that cytokines play an important role in the pathological development of laminitis. Cytochrome P450 (CYP450), also known as mono-oxygenase, drug-metabolizing enzyme, and multifunctional oxidase, is an important metabolic enzyme and involves in the metabolism of many endogenous and exogenous compounds [32]. Its three major subfamilies, CYP1, CYP2 and CYP3, play an important role in the metabolism and detoxification process [33]. The inflammation down-regulated the expressions of different subfamilies of CYP450 enzymes, leading to alterations in the pharmacokinetics of xenobiotics [34, 35]. Multiple studies have shown a rapid down-regulation of hepatic CYPs following administration of LPS [36, 37]. By RT-PCR method, our results proved that the expressions of CYP3A4 and CYP1A1 obviously decreased with the treatment of 1, 5, 10 μg/mL LPS. All above results demonstrated that the LPS-induced hoof dermal cells inflammatory model was established successfully.

At present, antibiotic injection is the main way for laminitis treatment [38]. However, with the abuse of antibiotics, the problems of drug resistance and side effects get worse. It is of great significance to find alternative herbs for the prevention and treatment of dairy hooves. Silymarin, which is an extract isolated from the fruit, rhizome and seeds of milk thistle, was proved to be a potent anti-inflammatory agent in the present study [15]. MTT assay was used to detect the toxic effects of silymarin, and the result showed that silymarin had no statistically significant effect on the viability of hoof dermal cells within the time and concentrations range determined in this experiment. In cigarette smoke-induced airway inflammatory model [39], bleomycin-induced pulmonary toxic model [40], and CCl4-induced liver damage model of mice [41], silymarin effectively inhibits the expressions of inflammatory cytokines such as TNF-α, IL-1β, IL-6 and IL-8. In the present study, it is further confirmed that the treatment with silymarin could decrease the levels of cytokines. Our data, and those of others, have demonstrated that the anti-inflammatory effect of silymarin might act through the NF-κB and MAPK signaling pathways [39, 42]. The expressions of CYP450s can be modulated by cytokines during inflammation, resulting in changes to the pharmacokinetics of medications. Therefore, CYP450 can be used for the development of new anti-inflammatory drugs and contribute to variability in drug efficacy or toxicity in inflammatory disease [43]. The present study showed that silymarin increased the expressions of CYP3A4 and CYP1A1, which indicated its effectiveness and securities as a potent anti-inflammatory agent. To sum up, silymarin plays an important role in protecting the LPS-induced inflammatory model of hoof dermal cells.

## Conclusions

10 μg/mL LPS can be used for inducing hoof dermal cells inflammatory model of dairy cows. 1 μg/mL silymarin can alleviate the inflammatory responses of hoof dermal cells caused by LPS stimulation in dairy cows. This study established a new experimental model for the study of laminitis in vitro, and can provide some useful information for the herbal medicine protection from animal hooves diseases.

## Abbreviations

CYP450: Cytochrome P450
DMEM: Dulbecco’s Modified Eagle Medium
DMSO: Dimethyl sulphoxide
EDTA: Ethylenediaminetetraacetic acid
ELISA: Enzyme-linked immunosorbent assay
FBS: Fetal bovine serum
HE staining: hematoxylin-eosin staining
IL-1β: Interleukin-1β
IR: inhibition ratio
ITS: Insulin-Transferrin-Selenium
LPS: lipopolysaccharides
MAPK: mitogen-activated protein kinase
MTT: Methylthiazolyldiphenyl-tetrazolium
NF-κB: Nuclear factor-κB
PBS: Phosphate buffered saline
TNF-α: Tumor necrosis factor-α
